# Supplementary material for: Thymosin β4 Regulates the Differentiation of Thymocytes by Controlling the Cytoskeletal Rearrangement and Mitochondrial Transfer of Thymus Epithelial Cells
Source: Int J Mol Sci. 2024 Jan 16;25(2):1088. doi: 10.3390/ijms25021088 (PMC10816181; doi:10.3390/ijms25021088)
Supplement: Supplementary file 1 [file ijms-25-01088-s001.zip › Table S1.pdf]

Table S1. Primer sequences of gene used in qPCR.

Gene	Primer sequences (5′ to 3′)
GAPDH (Mus)	Forward: GGCTGCCCAGAACATCAT<br>Reverse: CGGACACATTGGGGGTAG
Tβ4 (Mus)	Forward: GTCTGACAAACCCGATATGGCTGAG<br>Reverse: TCGCCAGCTTGCTTCTCTTGTTC
VCAM-1 (Mus)	Forward: CTCTTACCTGTGCGCTGTGA<br>Reverse: GACAGGTCTCCCATGCACAA
ICAM-1 (Mus)	Forward: GTGATGCTCAGGTATCCATCCA<br>Reverse: CACAGTTCTCAAAGCACAGCG
CD62L (Mus)	Forward: TACATTGCCCAAAAGCCCTTAT<br>Reverse: CCTCCTTGGACTTCTTGTTGTT
SPL (Mus)	Forward: GGTGTATGAGCTTATCTTCCAGC<br>Reverse: CTGTTGTTCGATCTTACGTCCA
S1P1 (Mus)	Forward: AGGGAACTTTGCGACTGAG<br>Reverse: GTTACAGCAAAGCCAGGTCAG
profilin-2 (Mus)	Forward: CTGAGGTGGGTGTCCTGGTT<br>Reverse: GCGTCTTGTCAGTCTTGGTG
e-cadherin (Mus)	Forward: CCAGAATTTGCCCAGCCCTA<br>Reverse: GTCCTCGTTCTTCAGGGCAA
